# Supplementary material for: Intrinsically disordered CsoS2 acts as a general molecular thread for α-carboxysome shell assembly
Source: Nat Commun. 2023 Sep 7;14:5512. doi: 10.1038/s41467-023-41211-y (PMC10484944; doi:10.1038/s41467-023-41211-y)
Supplement: Supplementary file 1 — Supplementary Information [file 41467_2023_41211_MOESM1_ESM.pdf]

Supplementary materials

for

Intrinsically disordered CsoS2 acts as a general molecular thread for α-carboxysome shell assembly

Tao Ni, Qiuyao Jiang, Pei Cing Ng, Juan Shen, Hao Dou, Yanan Zhu, Julika Radecke, Gregory F. Dykes, Fang Huang, Lu-Ning Liu, Peijun Zhang

List of Supplementary materials

Supplementary Table 1-3
Supplementary Figure 1 – 11

Supplementary Table 1. Cryo-EM data collection, refinement and validation statistics

	S2/4A-1A (mini-shell-2)		4A-1A (mini-shell-1)	
**Data collection and processing**	*T* = 9	*T* = 4	*T* = 3	*T* = 4
Magnification (nominal)	105k	105k	130k	130k
Voltage (kV)	300	300	300	300
Detector	Gatan K3	Gatan K3	Falcon 4	Falcon 4
Energy filter	BioQuantum 20 eV slit	BioQuantum 20 eV slit	Selectris X 10 eV slit	Selectris X 10 eV slit
Electron exposure (e–/Å^2)	44	44	40	40
Defocus range (μm)	-1.0 to -3.0	-1.0 to -3.0	-1.0 to -3.0	-1.0 to -3.0
Pixel size (Å)	0.831	0.831	0.92	0.92
Symmetry imposed	I1	I1	I1	I1
Initial particle images (no.)	143769	396380*	201078	18088
Images (no.)	20238	20238	5124	5124
Final particle images (no.)	123500	13515	159149	2011
Map resolution (Å)				
FSC threshold	0.143	0.143	0.143	0.143
Map resolution (Å)	1.86	2.67	2.79	3.54
**Refinement**				
Initial model used (PDB code)	2ewh, 2rcf	2ewh, 2rcf	2ewh, 2rcf	
Model resolution (Å)	2.4	2.63	2.9	
FSC threshold	0.5	0.5	0.5	
Map sharpening *B* factor (Å^2)	-80.6	-61.8	-95.4	
Model composition				
Non-hydrogen atoms	7171	2624	1953	
Protein residues	937	360	267	
Ligands				
B factors (Å^2)				
Protein	42.3	14.56	18.49	
Ligand				
R.m.s. deviations				
Bond lengths (Å)	0.003	0.003	0.004	
Bond angles (°)	0.611	0.661	0.616	
Validation				
MolProbity score	1.29	1.42	1.32	
Clashscore	5.40	7.64	5.90	
Poor rotamers (%)	0.76	0		
Ramachandran plot				
Favored (%)	99.34	99.14	99.23	
Allowed (%)	0.55	0.86	0.77	
Disallowed (%)	0.11	0	0	
Accession codes	EMD-15801 PDB 8B12	EMD-15799 PDB 8B11	EMD-15798 PDB 8B0Y	EMD-15792

* Autopicking selected a large amount of junk particles that were subsequently removed through 2D classification.

Supplementary Table 2. Cryo-EM data collection, refinement and validation statistics of mini-shell mutants

	S2-C1/4A-1A (mini-shell-4)					
**Data collection and processing**	*T* = 9	*T* = 7	*T* = 7 *Q* = 6	*T* = 4 *Q* = 6 class 1	*T* = 4 *Q* = 6 class 2	*T* = 4
Magnification (nominal)	105k	105k	105k	105k	105k	105k
Voltage (kV)	300	300	300	300	300	300
Detector	Gatan K3	Gatan K3	Gatan K3	Gatan K3	Gatan K3	Gatan K3
Energy filter	BioQuantum 20 eV slit	BioQuantum 20 eV slit	BioQuantum 20 eV slit	BioQuantum 20 eV slit	BioQuantum 20 eV slit	BioQuantum 20 eV slit
Electron exposure (e–/Å^2)	40	40	40	40	40	40
Defocus range (μm)	-0.9 to -2.5	-0.9 to -2.5	-0.9 to -2.5	-0.9 to -2.5	-0.9 to -2.5	-0.9 to -2.5
Pixel size (Å)	0.831	0.831	0.831	0.831	0.831	0.831
Symmetry imposed	I1	I1	D3	D5	D5	I1
Initial particle images (no.)	143645	143645	143645	143645	143645	143645
Images (no.)	13151	13151	13151	13151	13151	13151
Final particle images (no.)	25158	7871	7464	7651	7875	84561
FSC threshold	0.143	0.143	0.143	0.143	0.143	0.143
Map resolution (Å)	2.04	3.29	3.17	2.65	2.69	2.02
Accession codes	EMD-15722	EMD-15720	EMD-15595	EMD-15723	EMD-15724	EMD-15719

Supplementary Table 2. Cryo-EM data collection, refinement and validation statistics of mini-shell mutants (continued)

	S2-C2/4A-1A (mini-shell-5)	S2-C3/4A-1A (mini-shell-6)				S2-Cm/4A-1A	
**Data collection and processing**	*T* = 4	*T* = 4	*T* = 4 - P	*T* = 3	*T* = 3 - P	*T* = 3	*T* = 4
Magnification (nominal)	105k	105k	105k	105k	105k	105k	105k
Voltage (kV)	300	300	300	300	300	300	300
Detector	Gatan K3	Gatan K3	Gatan K3	Gatan K3	Gatan K3	Gatan K3	Gatan K3
Energy filter	BioQuantum 20 eV slit	BioQuantum 20 eV slit	BioQuantum 20 eV slit	BioQuantum 20 eV slit	BioQuantum 20 eV slit	BioQuantum 20 eV slit	BioQuantum 20 eV slit
Electron exposure (e–/Å^2)	40	40	40	40	40	40	40
Defocus range (μm)	-0.9 to -2.5	-0.9 to -2.5	-0.9 to -2.5	-0.9 to -2.5	-0.9 to -2.5	-0.9 to -2.5	-0.9 to -2.5
Pixel size (Å)	0.831	0.831	0.831	0.831	0.831	0.831	0.831
Symmetry imposed	I1	I1	I1	I1	I1	I1	I1
Initial particle images (no.)	377408*	101598	101598	101598	101598	78569	78569
Images (no.)	3238	9060	9060	9060	9060	3683	3683
Final particle images (no.)	38116	17029	4404	52521	21591	68634	9935
FSC threshold	0.143	0.143	0.143	0.143	0.143	0.143	0.143
Map resolution (Å)	2.26	2.39	3.38	2.31	2.68	2.22	2.65
Accession codes	EMD-15611	EMD-15758	EMD-15759	EMD-15760	EMD-15761	EMD-15834	EMD-15762

* Autopicking selected a large amount of junk particles that were subsequently removed through 2D classification.

Supplementary Table 3. ssDNA oligonucleotides used in this study. The overlapping sequences for Gibson assembly are underlined.

Primer	Sequence (5' to 3')	Description
pBAD-S4A-S1A-FW	TTTTGGGCTAACAGGAGGAATTAACTTTGAGCGTTCAGGCGCA	Construction of mini-shell-1
CsoS4A-RV	TTACTCACCATTCCACTG	
CsoS1A-FW	TATTGATCAGTGGAATGGTGAGTAAGGATTGGGAAAGACGAAC	
pBAD-S4A-S1A-RV	TTTTGTTCTACGTAAGCTTCGAATTTTAGGCTTGTGGCGCCTT	
pBAD-S2-S4A-S1A-FW	TTTTGGGCTAACAGGAGGAATTAACATGGGGTCAAACATGCCT	Construction of mini-shell-2
CsoS2-RV	TCAACCGCGCGCGCCGCC	
CsoS4A-S1A-FW	TTACTCCGGCGGCGCGCGCGGTTGATTTGAGCGTTCAGGCGCA	
pBAD-CsoS2-S4A-S1A-RV	TTTTGTTCTACGTAAGCTTCGAATTTTAGGCTTGTGGCGCCTT	
pBAD-S2-S1A-FW	TTTTGGGCTAACAGGAGGAATTAACATGGGGTCAAACATGCCT	Construction of mini-shell-3
CsoS2-RV	TCAACCGCGCGCGCCGCC	
CsoS1A-FW	TTACTCCGGCGGCGCGCGCGGTTGAGGATTGGGAAAGACGAAC	
pBAD-S2-S1A-RV	TTTTGTTCTACGTAAGCTTCGAATTTTAGGCTTGTGGCGCCTT	
pBAD-S2-C1-S4A-S1A-FW	TTTTGGGCTAACAGGAGGAATTAACATGCTTCCCACTAGTCCACGC	Construction of mini-shell-4
pBAD-S2-C2-S4A-S1A-FW	TTTTGGGCTAACAGGAGGAATTAACATGCACGCTGCGCGCGAGTTG	Construction of mini-shell-5
pBAD-S2-C3-S4A-S1A-FW	TTTTGGGCTAACAGGAGGAATTAACATGAATGCGCGTGTGGTCGAA	Construction of mini-shell-6
pBAD-S2-Cm-S4A-S1A-FW	GGAGGAATTAACCATGGATCCGAGCATGTCAACTGAACAATCATTGACC	Construction of S2-Cm mini-shell
S2-Cm-RV	GATATCCTGCGCCTGAACGCTCAAATCAACCGCGCGCGCCGCCGGA	
CsoS4A-S1A-FW-2	GGCGTCCGGCGGCGCGCGCGGTTGATTTGAGCGTTCAGGCGCAGGA	
pBAD-S4A-S1A-RV-2	TTTTGTTCTACGTAAGCTTCGAATTTTAGGCTTGTGGCGCCTTAGG	

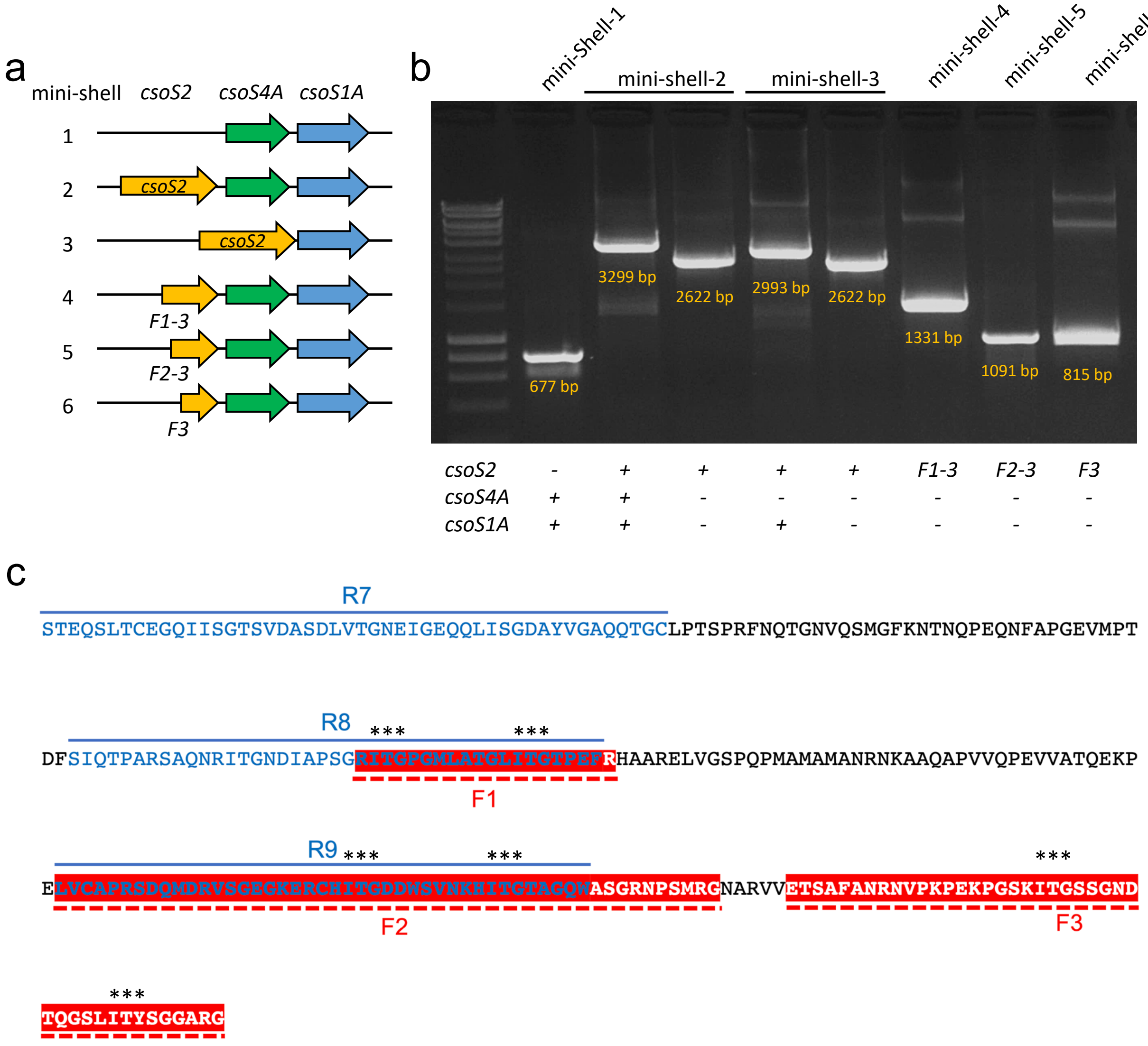

Supplementary Fig. 1 | Construction of the different mini-shell forms. (a) The genetic arrangements of mini-shell-1 to mini-shell-6 constructs generated in this study. (b) PCR verification of the mini-shell constructs using the primers listed in Supplementary Table 3. The genes in PCR products are indicated at the bottom of the gel image. Two sets of primers are used in the mini-shell 2 and 3 constructs. The sizes (bp) of PCR products are labelled in orange. This experiment was repeated and confirmed by further plasmid sequencing results. (c) The protein sequence of CsoS2 C-terminal domain. The three interaction fragments in the C-terminal region (F1, F2, F3) newly identified in the *T* = 9 shell are shown in red. The three additional repeats in the C-terminal region (R7, R8, R9) previously identified (1) are represented in blue. *** indicates the I(V)TG motif, which was replaced by AAA in the CsoS2-Cm mutant.

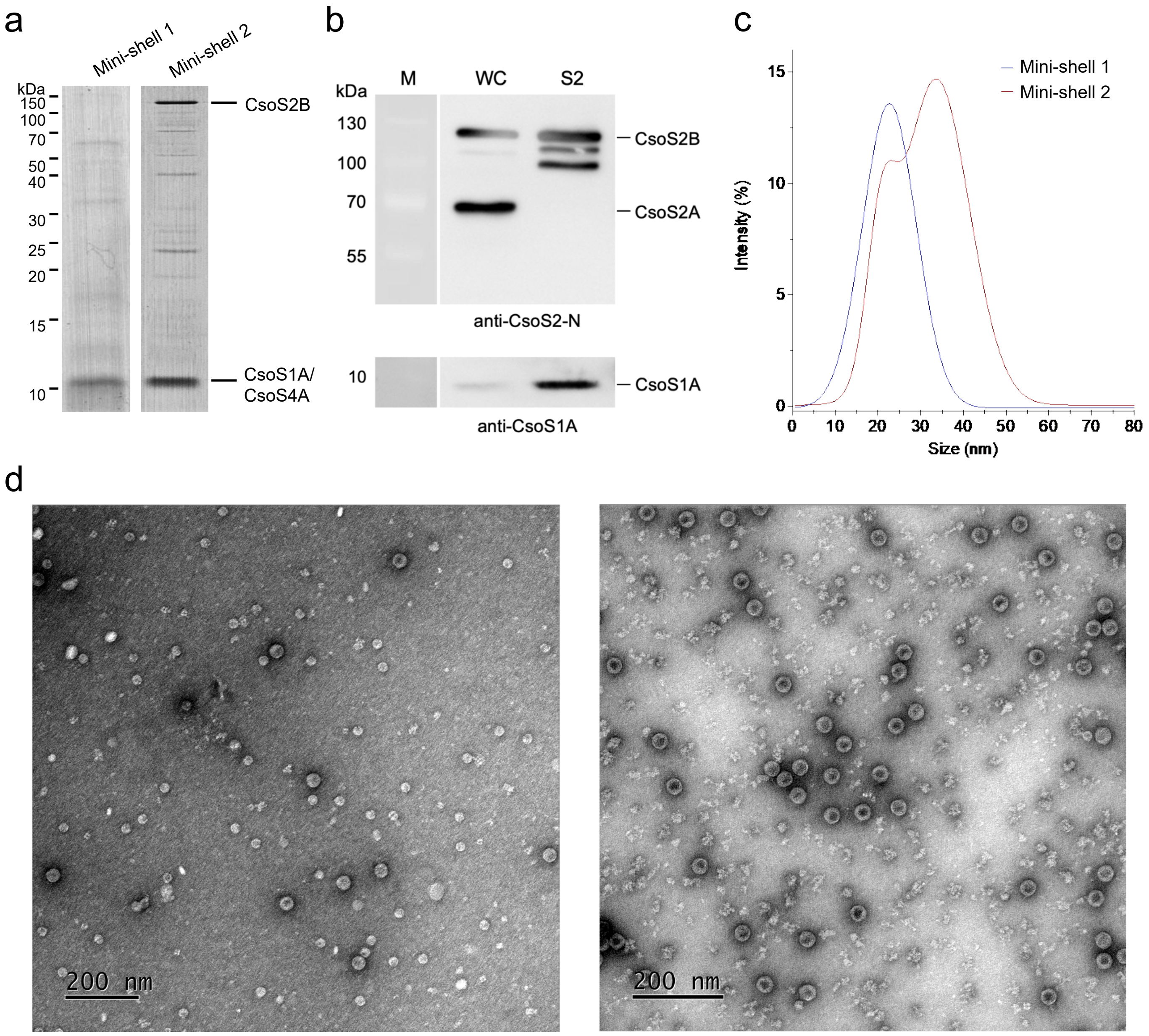

Supplementary Fig. 2 | Characteristics of shells generated from mini-shell 1 (CsoS4A-CsoS1A) and mini-shell 2 (CsoS2-CsoS4A-CsoS1A) constructs. (a) SDS-PAGE results revealed the major protein components of purified shells from mini-shell-1 and mini-shell-2. The purification was performed at least three times and a representative purification result is shown. (b) Immunoblot analysis using anti-CsoS2 antibody (GenScript, USA) revealed that both CsoS2A and CsoS2B were expressed in the *E. coli* mini-shell 2 construct, but only CsoS2B was incorporated into the mini-shells and CsoS2A was not detectable. WC: whole cell lysate of the CsoS2-CsoS4A-CsoS1A mini-shell 2 construct; S2: isolated CsoS2-CsoS4A-CsoS1A mini-shells. The experiment was performed at least three times and a representative blot is shown. (c) Dynamic light scattering (DLS) analysis of shell sizes from mini-shell 1 and mini-shell 2. (d) Electron microscopy (EM) images of negatively stained purified shells from mini-shell 1 and mini-shell 2. The experiments were repeated at least three times with two representative micrographs shown here.

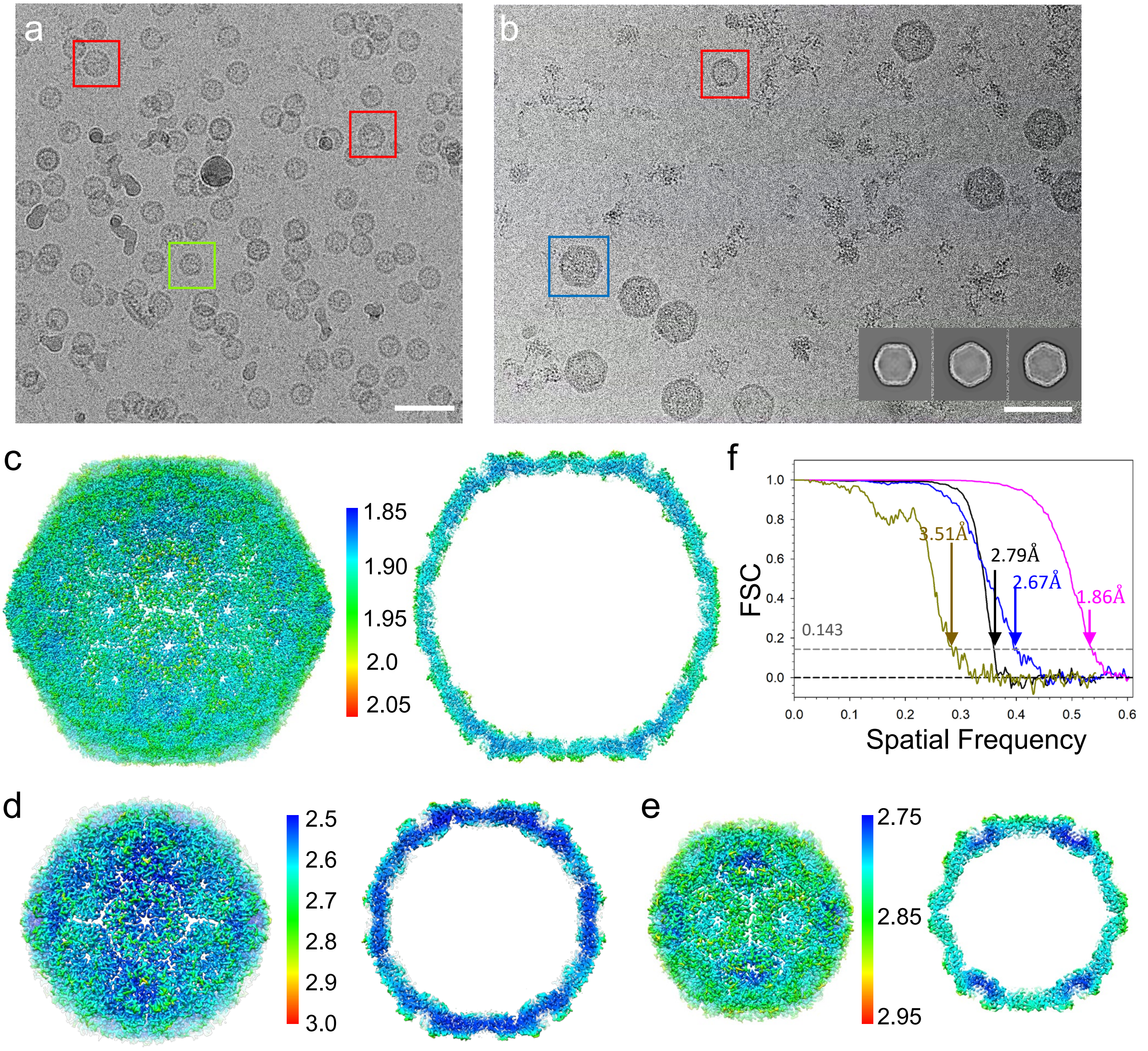

Supplementary Fig. 3 | CryoEM data processing. (a-b) Representative micrographs and 2D class averages of shells produced from mini-shell 1 (a) and mini-shell 2 (b), respectively. Boxed particles have different sizes: blue, large shells (T = 9); red, medium shell (T = 4); and green, small shell (T = 3). The cryo-EM imaging and analysis for these two samples were performed only once. Scale bars: 50 nm. (c-e) CryoEM maps of T = 9 (c), T = 4 (d) and T = 3 (e), shown in top view (left) and central slice (right). Maps are coloured according to their local resolutions. (f) Fourier Shell Correlation (FSC) of shells, T = 9 in magenta, T = 4 in blue (from mini-shell 2) and brown (from mini-shell 1), and T = 3 in black, with resolutions indicated at FSC=0.143 cut-off.

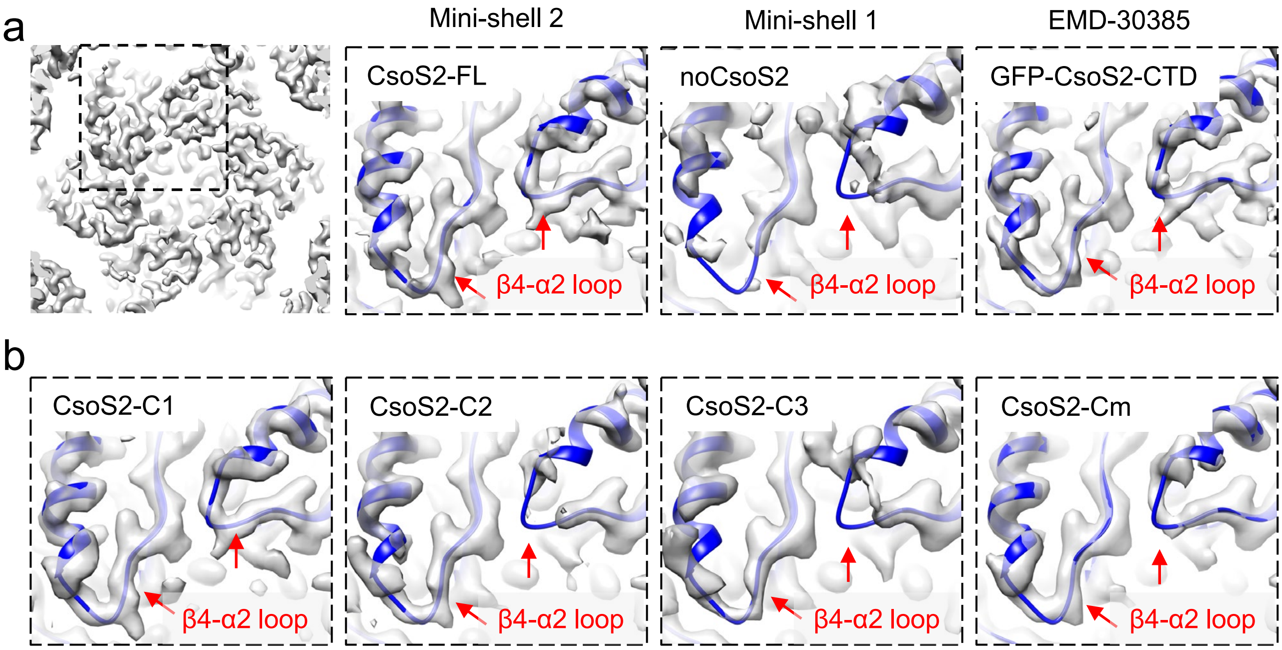

Supplementary Fig. 4 | Comparison of T = 4 shells with and without CsoS2 from different mini-shell constructs. (a) Comparison of T = 4 shell hexamer maps (gray) from mini-shell-1, mini-shell-2, and a GFP-CsoS2-CTD/4A-1A (EMD-30385). The major difference in hexamer density is located in the loop between β4 and α2 in S1A (red arrows), which could not be resolved in the shell without CsoS2. (b) Density maps of T = 4 shell hexamers from different CsoS2 truncation and I(V)TG mutation constructs. The map contour level is set to 4.5σ for all the maps except EMD-30385 (2σ).

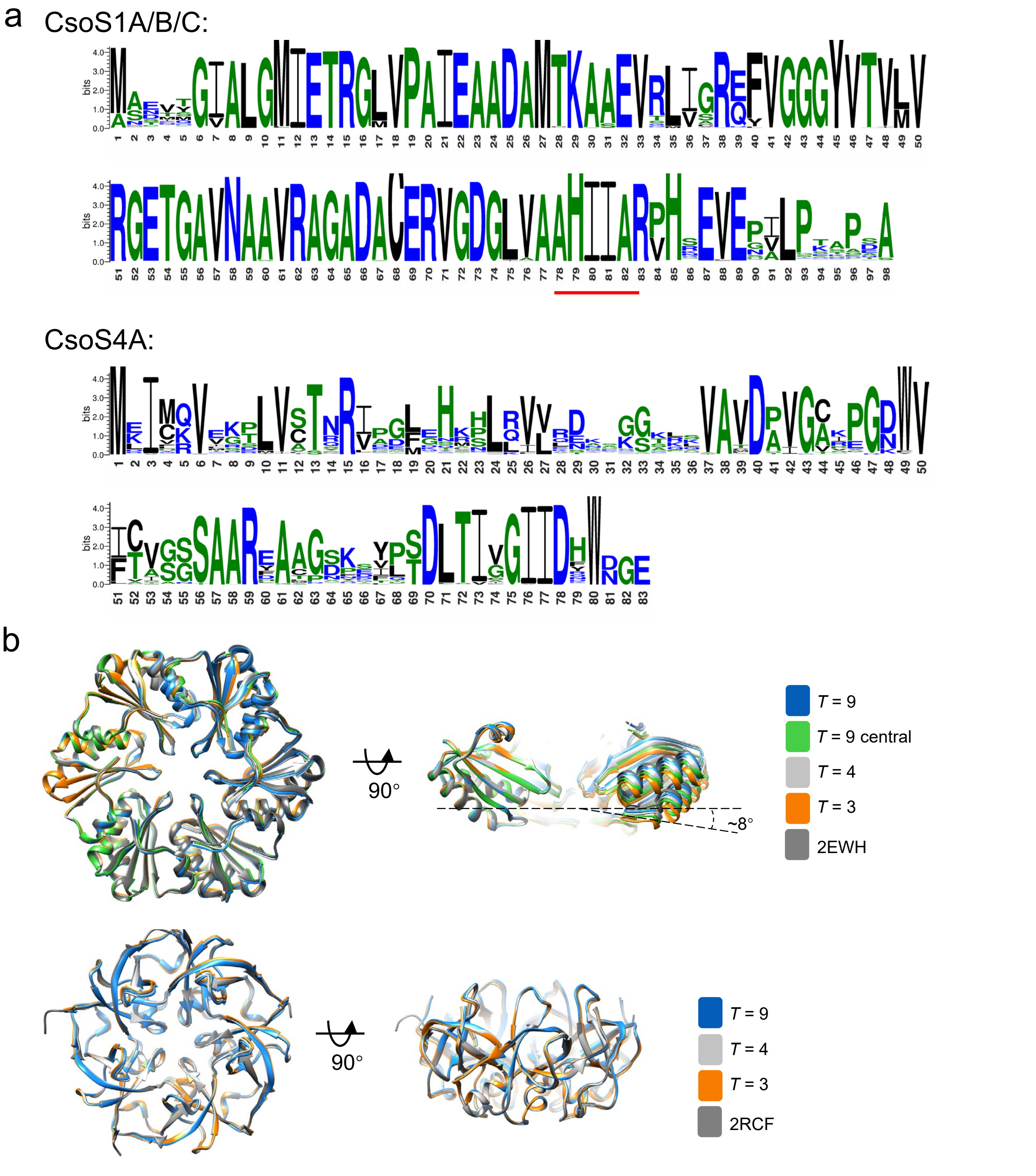

Supplementary Fig. 5 | Sequence and structural analysis of CsoS1A and CsoS4A. (a) Conservation of CsoS1A/B/C (990 sequences) and CsoS4A (970 sequences) from the Uniprot database, presented using Weblogo. CsoS1A and CsoS1C have only two residues that are distinct from each other, and CsoS1A and CsoS1B are 91% identical. Only 40% similarity was found between CsoS4A and CsoS4B. The red line indicates the conserved β-strand interacting with the I(V)TG motif of CsoS2. (b) Structural comparison of CsoS1A hexamer (top) and CsoS4A pentamers (bottom) from mini-shell assemblies and X-ray crystallography structures in two orthogonal views, indicating very little deviations among these structures. Two quasi-equivalent hexamers from T = 9 shell are shown in blue (close to pentamer) and green (at the 3-fold), respectively (see Figure 2A). The hexamers in the T = 3 shell have the maximum curvature (~ 8°) compared with the crystal structure (PDB: 2EWH).

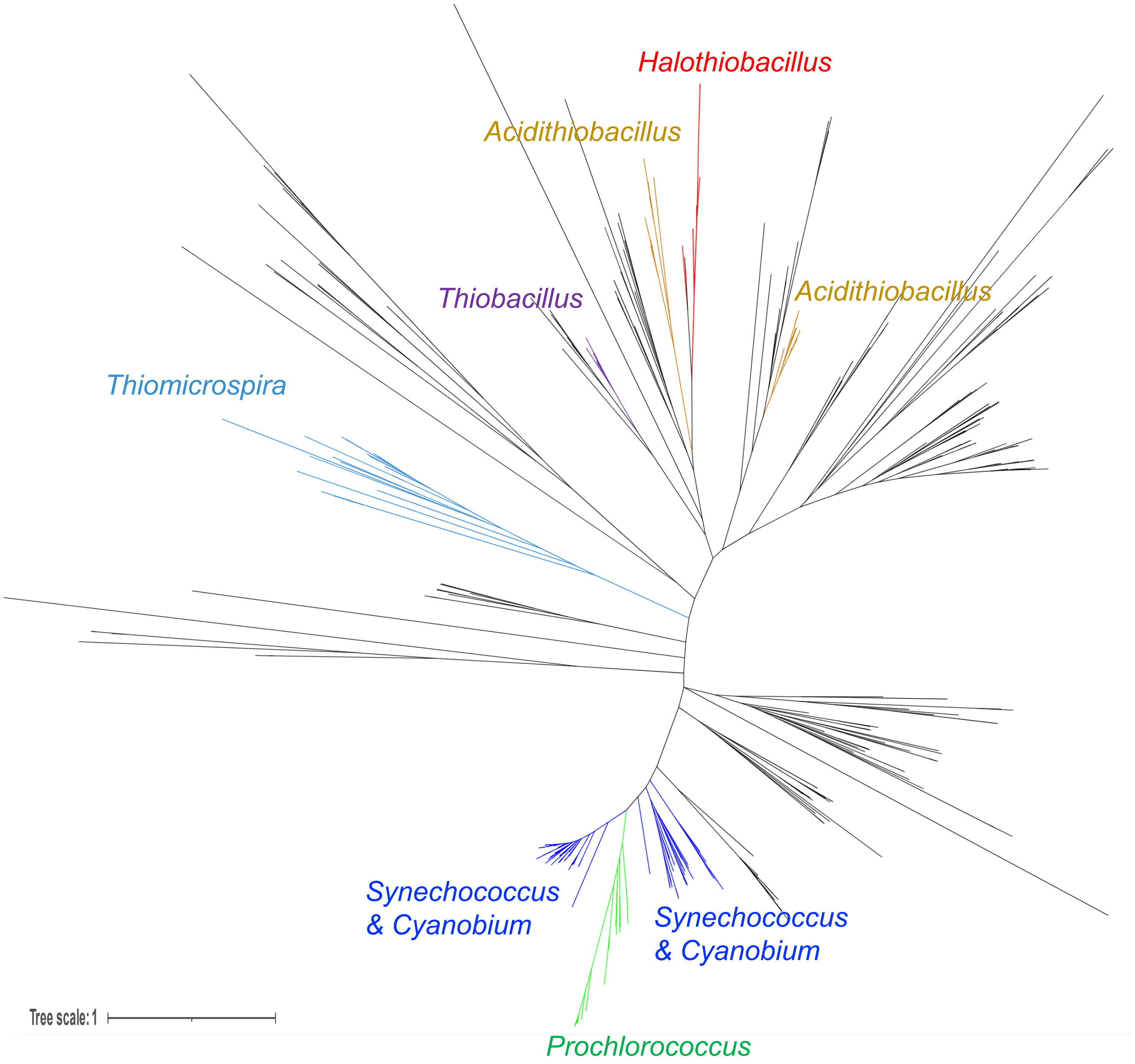

Supplementary Fig. 6 | Maximum-likelihood phylogenetic tree of CsoS2. Among the 395 bacterial species containing CsoS2 homologs, clades of *Halothiobacillus, Thiobacillus, Thiomicrospira*, *Acidithiobacillus, Prochlorococcus, Synechococcus, and Cyanobium* are colored in red, violet, yellow, lollipop, green, and blue, respectively. Scale bar, 1 substitution per site.

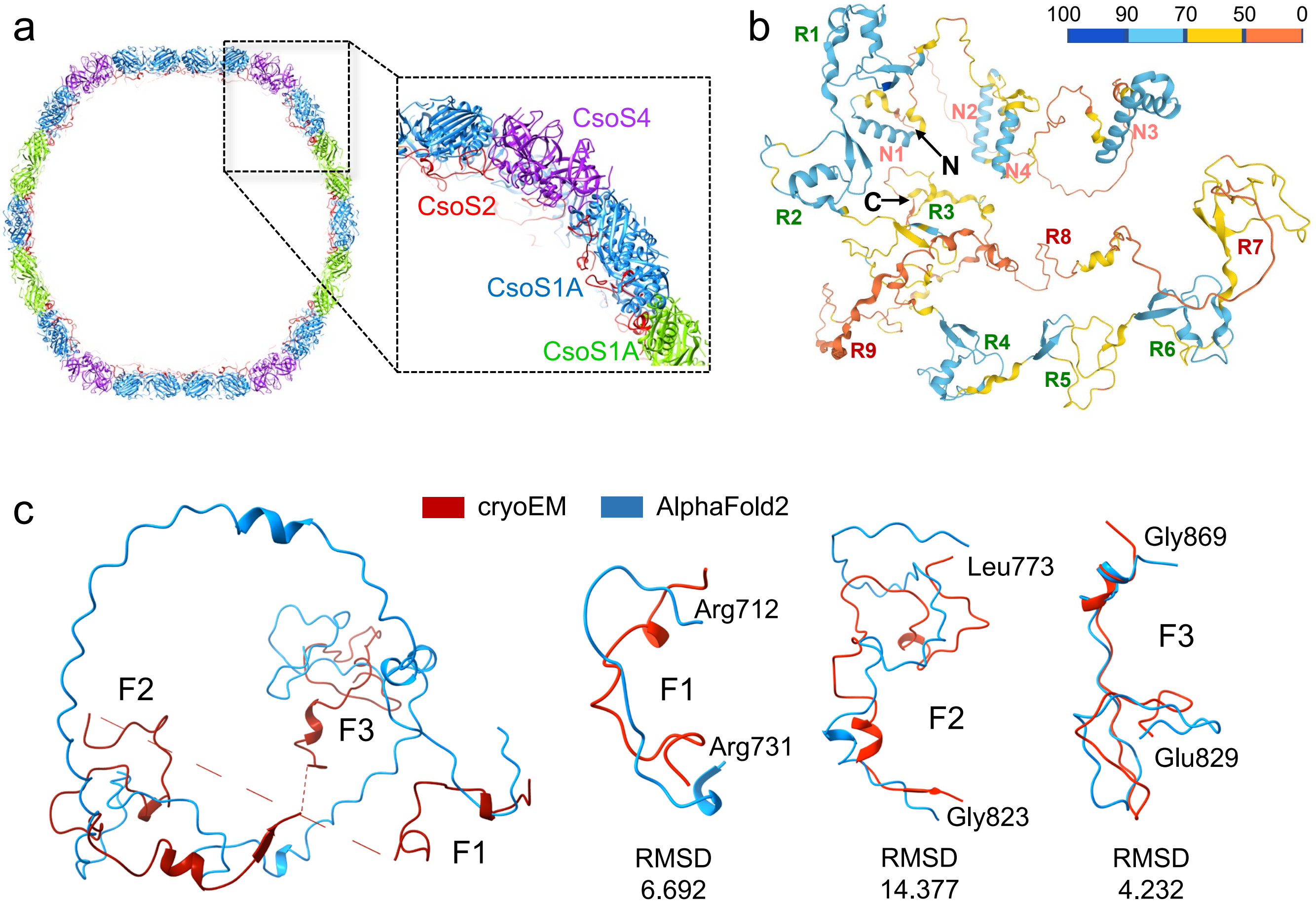

Supplementary Fig. 7 | Identification and characterization of CsoS2 C-terminal fragments in mini-shell 2. (a) Localization of CsoS2 at the inner surface of *T* = 9 shell assembly, shown as a central slice. Shell proteins are coloured the same as in Figure 1d. Inset shows a close-up view. (b) AlphaFold2 structure prediction of CsoS2. The predicted model is coloured according to model confidence scores (pLDDT) as indicated. The N- and C- termini and domains are labelled. (c) Overlay of CsoS2 structures from AlphaFold2 prediction (blue) and cryoEM (red). The structure of three individual fragments resolved by cryoEM and predicted from AlphaFold2 are overlaid on the right.

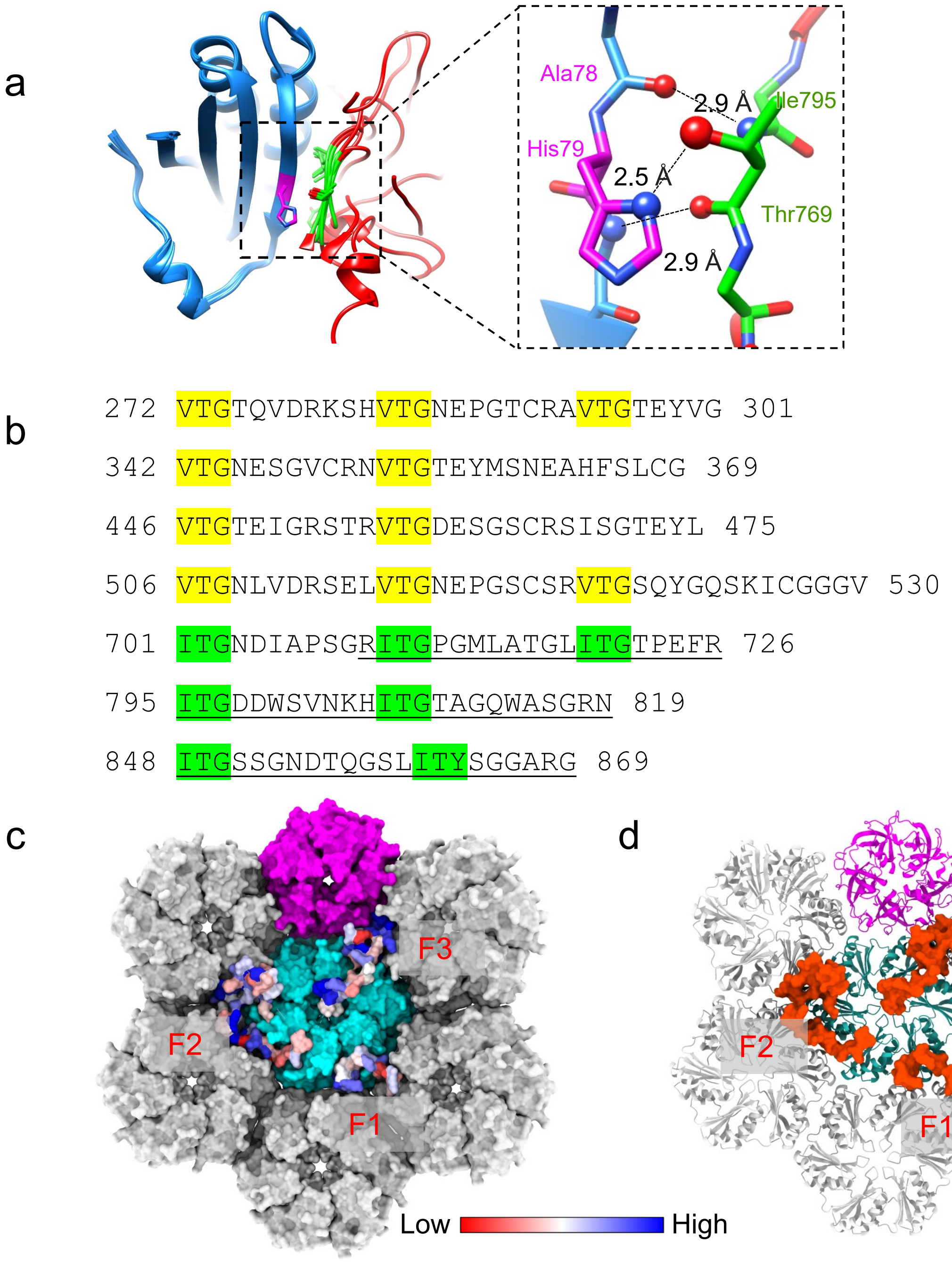

Supplementary Fig. 8 | Conservation of CsoS2 C-terminal domain. (a) Hydrogen bond network between CsoS1A (blue) and a CsoS2 [IV]TG motif (red), mediated by both main-chain and side-chain hydrogen bonds. A closeup view of one of the [IV]TG motif interfaces (I795-T796-G797 in F2) is shown on the right. (b) [IV]TG motifs in *H. neapolitanus* CsoS2. The [IV]TG motifs in the Middle region and C-terminal domain are highlighted in yellow and green, respectively. The range of amino acid residues is labeled. (c) Surface rendering of the structure of CsoS2 C-terminal domain in complex with CsoS1A and CsoS4A in *T* = 9 shell. Only one copy of CsoS2 molecule is shown; the symmetry-related copies are removed for clarity. The CsoS1A hexamers are colored in cyan and gray, and the CsoS4A pentamer in purple. CsoS2 surface is colored according to conservation scores, ranging 1 to 10 (red to blue). The conservation score of CsoS2 fragments is calculated with ConSurf server (https://consurf.tau.ac.il/consurf_index.php). (d) Ribbon representation of the same structure shown in (b), with CsoS1A hexamers in gray/sea green, CsoS4A pentamer in purple and CsoS2 in red.

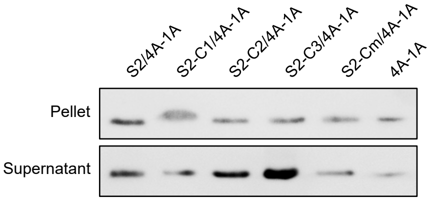

Supplementary Fig. 9 | Western blot of mini shell constructs with different CsoS2 mutants. The assembled shell (in the Pellet after 30% sucrose cushion ultracentrifugation) and free shell proteins (in the Supernatant after 30% sucrose cushion ultracentrifugation) were probed with anti-CsoS1A antibody. The ratios of assembled shell and free shell proteins are compared among the constructs (see Fig.4b). The immunoblot was normalised against the amount of assembled mini-shells across the constructs.

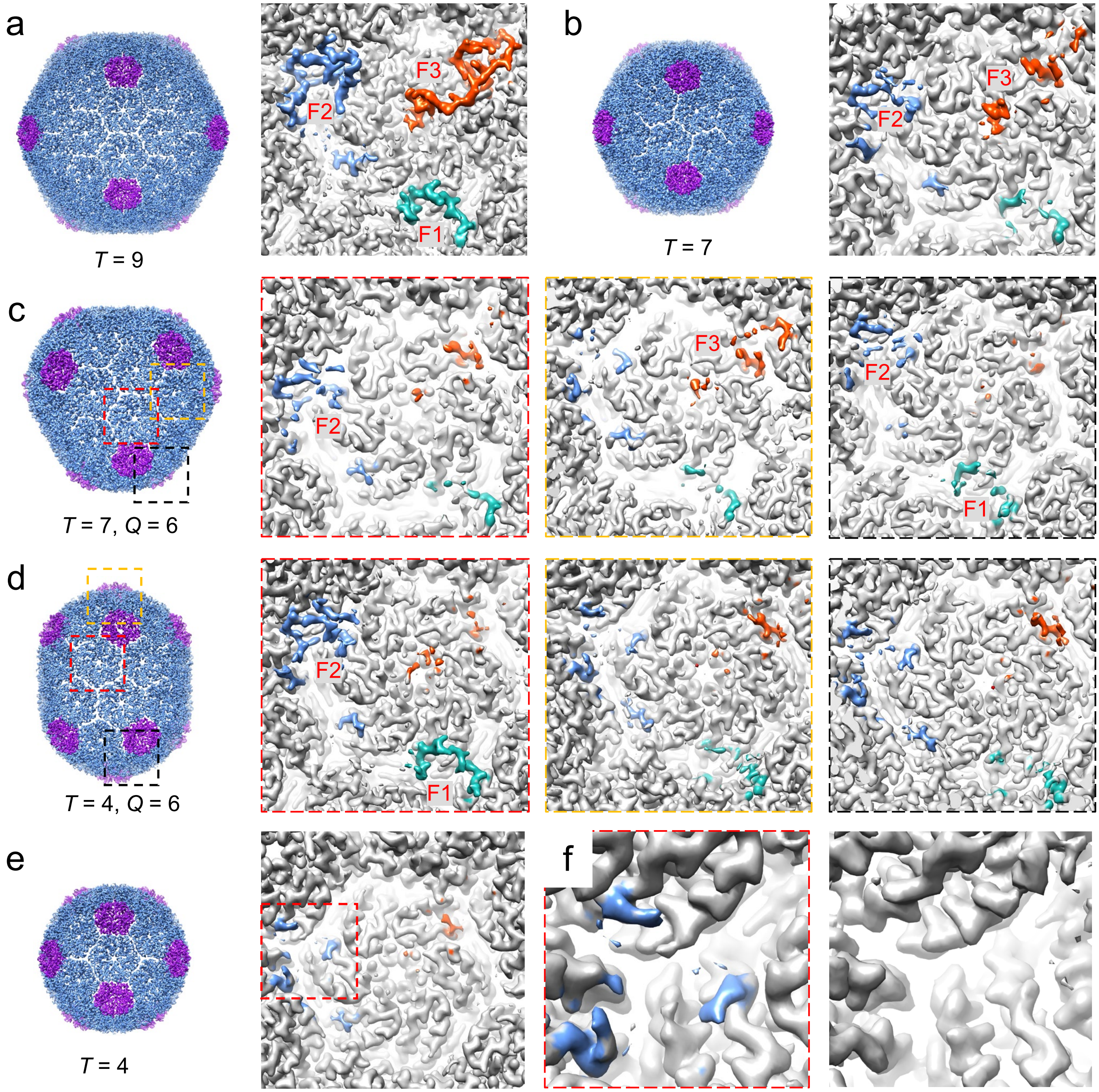

Supplementary Fig. 10 | CsoS2 density in shell assemblies from the mini-shell 4 construct containing S2-C1). (a) *T* = 9 shell with a close-up view of CsoS2 F1, F2 and F3 fragments, coloured in light sea green, blue and orange, respectively. (b) *T* = 7 shell with a close-up view of CsoS2 F1, F2 and F3 fragments, where only F2 and F3 fragments can be assigned confidently, albeit with weaker density. (c) *T* = 7, *Q* = 6 shell with close-up views of three quasi-equivalent interfaces. The densities corresponding to [IV]TG motifs can be observed. (d) *T* = 4, *Q* = 6 shell with close-up views of three quasi-equivalent interfaces. CsoS2 densities are coloured according to their respective fragments. (e) *T* = 4 shell with a close-up view. (f) Comparison of *T* = 4 shells from mini-shell 2 (left) and mini-shell 1 (right). The densities of CsoS2 are colored in blue. Only the residual densities corresponding to [IV]TG motifs can be observed.

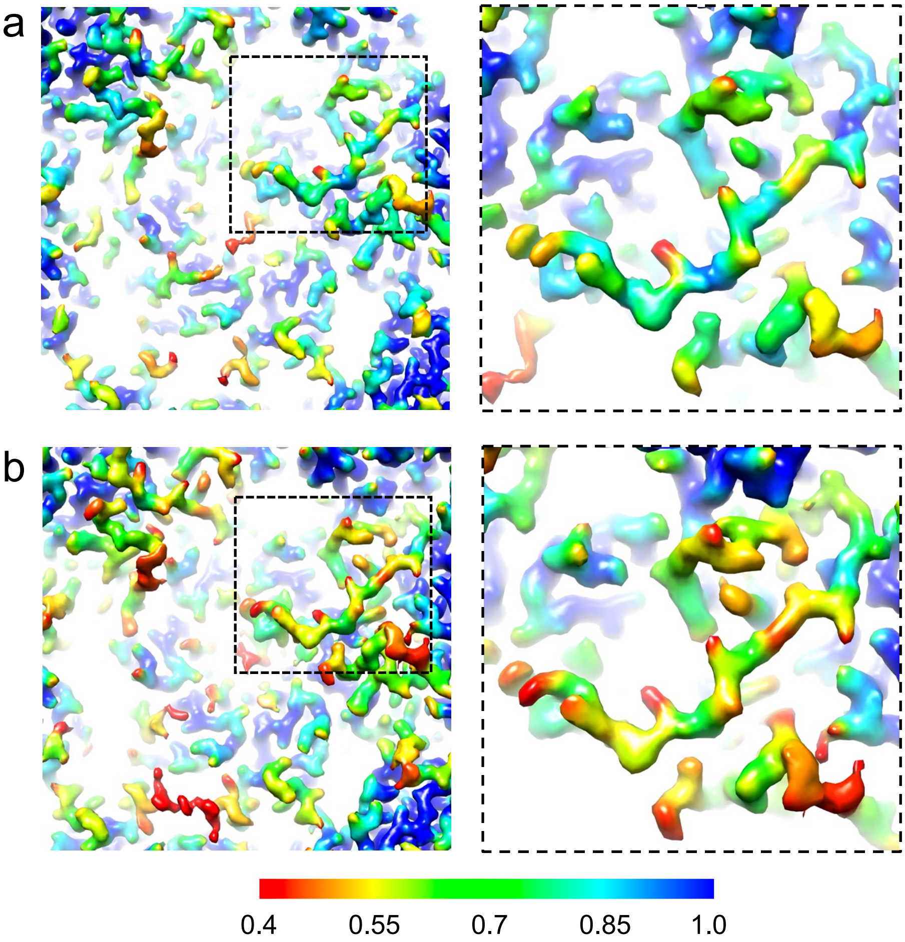

Supplementary Fig. 11 | Quantification of CsoS2 occupancy by Occupy. (a) Occupancy map of T=9 shell from the full-length CsoS2 construct, with a F3 fragment zoomed in (right). (b) Occupancy map of T=9 shell from the truncated C1 construct, with a F3 fragment zoomed in (right). Density map of a hexamer from the T=9 shell colored by occupancy from 0.4 to 1 (red to green). F3 fragment in T=9 C1 construct has lower occupancy.
