# Supplementary material for: Intrinsically disordered CsoS2 acts as a general molecular thread for α-carboxysome shell assembly
Source: Nat Commun. 2023 Sep 7;14:5512. doi: 10.1038/s41467-023-41211-y (PMC10484944; doi:10.1038/s41467-023-41211-y)
Supplement: Supplementary file 3 — Reporting Summary [file 41467_2023_41211_MOESM3_ESM.pdf]

nature portfolio

Corresponding author(s): Peijun Zhang

Last updated by author(s): Aug 14, 2023

Reporting Summary

Nature Portfolio wishes to improve the reproducibility of the work that we publish. This form provides structure for consistency and transparency in reporting. For further information on Nature Portfolio policies, see our Editorial Policies and the Editorial Policy Checklist.

Statistics

For all statistical analyses, confirm that the following items are present in the figure legend, table legend, main text, or Methods section.

n/a	Confirmed	
☐	☒	The exact sample size (*n*) for each experimental group/condition, given as a discrete number and unit of measurement
☒	☐	A statement on whether measurements were taken from distinct samples or whether the same sample was measured repeatedly
☒	☐	The statistical test(s) used AND whether they are one- or two-sided<br>*Only common tests should be described solely by name; describe more complex techniques in the Methods section.*
☒	☐	A description of all covariates tested
☒	☐	A description of any assumptions or corrections, such as tests of normality and adjustment for multiple comparisons
☐	☒	A full description of the statistical parameters including central tendency (e.g. means) or other basic estimates (e.g. regression coefficient) AND variation (e.g. standard deviation) or associated estimates of uncertainty (e.g. confidence intervals)
☒	☐	For null hypothesis testing, the test statistic (e.g. *F*, *t*, *r*) with confidence intervals, effect sizes, degrees of freedom and *P* value noted<br>*Give P values as exact values whenever suitable.*
☒	☐	For Bayesian analysis, information on the choice of priors and Markov chain Monte Carlo settings
☒	☐	For hierarchical and complex designs, identification of the appropriate level for tests and full reporting of outcomes
☒	☐	Estimates of effect sizes (e.g. Cohen's *d*, Pearson's *r*), indicating how they were calculated

Our web collection on statistics for biologists contains articles on many of the points above.

Software and code

Policy information about availability of computer code

Data collection	cryoEM data were collected using EPU (v2) which is part of the ThermoFisher electron microscope system.
Data analysis	cryoEM data processing: relion (v3 and v4) and cryosparc (v3 and v4) (available and referenced in methods).<br>Model building: AlphaFold2 (alphaflod colab), Coot (v0.8.9.2), Chimera (v1.15), Phenix (v1.19.2-4158), PyMOL (v2.0) (available and referenced in methods). Data plotting: GraphPad Prism 9 (GraphPad Software, San Diego, California USA, www.graphpad.com). Fiji software for negative staining EM image analysis.

For manuscripts utilizing custom algorithms or software that are central to the research but not yet described in published literature, software must be made available to editors and reviewers. We strongly encourage code deposition in a community repository (e.g. GitHub). See the Nature Portfolio guidelines for submitting code & software for further information.

Data

Policy information about availability of data

All manuscripts must include a data availability statement. This statement should provide the following information, where applicable:

- Accession codes, unique identifiers, or web links for publicly available datasets
- A description of any restrictions on data availability
- For clinical datasets or third party data, please ensure that the statement adheres to our policy

The cryo-EM density maps and corresponding atomic models have been deposited in the EMDB and PDB, respectively. The accession codes are listed as follows: (1)

CsoS4A and CsoS1A (mini-shell-1 construct): EMD-15798 and PDB 8B0Y for T = 4 shell, EMD-15792 for T = 3 shell; (2) Full-length CsoS2 with CsoS4A and CsoS1A (mini-shell-2 construct): EMD-15801 and PDB 8B12 for T = 9 shell, EMD-15799 and PDB 8B11 for T = 4 shell; The raw micrographs have been deposited to EMPIAR with accession code of EMPIAR-11559. (3) Truncated CsoS2 with F1-F3 fragments (mini-shell-4 construct): EMD-15722 (T = 9), EMD-15720 (T = 7), EMD-15595 (T = 7, Q = 6), EMD-15723 (T = 4, Q = 6 class 1), EMD-15724 (T = 4, Q = 6 class 2), EMD-15719 (T = 4); The raw micrographs have been deposited to EMPIAR with accession code of EMPIAR-11560. (4) Truncated CosS2 with F2-F3 fragments (mini-shell-5 construct): EMD-15611 for T = 4 shell; (5) Truncated CosS2 with F3 fragment (mini-shell-6 construct): EMD-15758 (T = 4 with pentamer), EMD-15759 (T = 4 without pentamer), EMD-15760 (T = 4 with pentamer) and EMD-15761 (T = 4 without pentamer); (6) CsoS2-C with [IV]TG mutant: EMD-15834 (T = 4) and EMD-15762 (T = 3).

Human research participants

Policy information about studies involving human research participants and Sex and Gender in Research.

Reporting on sex and gender	Not applicable
Population characteristics	Not applicable
Recruitment	Not applicable
Ethics oversight	Not applicable

Note that full information on the approval of the study protocol must also be provided in the manuscript.

Field-specific reporting

Please select the one below that is the best fit for your research. If you are not sure, read the appropriate sections before making your selection.

☒ Life sciences ☐ Behavioural & social sciences ☐ Ecological, evolutionary & environmental sciences

For a reference copy of the document with all sections, see nature.com/documents/nr-reporting-summary-flat.pdf

Life sciences study design

All studies must disclose on these points even when the disclosure is negative.

Sample size	For cryo-EM SPA, sample sizes were those required for the resolution. We typically aim to achieve resolution of resulting structures beyond 3.5 angstrom from one dataset, which is achievable from each single dataset reported in the work, typically collected in one or two-day cryo-EM session. The details of datasets, including sample sizes, are listed int Supplementary Table 1 and 2.
Data exclusions	During Cryo-EM SPA study, the particles were autopicked either with a template or by a density threshold, which is a standard approach in Relion and cryoSPARC. 2D and 3D classifications were performed to remove the junk particles in order to generate higher-resolution density maps.
Replication	Fro cryoEM SPA study, two randomly divided half datasets were processed independently and combined to give rise to the final structures. The resolution of the structure is assessed by comparing the two independent maps. For each cryo-EM dataset, it was collected from one or two sessions using the same sample. There are variations between batches of sample purification, and only the best sample screened from cryo-EM grids were collected to obtain a complete dataset.<br>For western blot experiment, at least three replication experiments were performed and quantified. One of the representative images were presented in the figure.
Randomization	For each dataset of Cryo-EM SPA study, the dataset were randomly divided into two half sets, as a standard approach implemented in Relion and cryoSPARC
Blinding	The cryo-EM SPA study including data collection and processing is not blinded. The mini-shell samples have a defined shape (icosahedral, oblate and prolate) compared with the other molecules and can be classified through the data processing pipeline. The contaminations that occasionally were included in the sample (such as ribosomes) were classified out and removed from data during processing.

Reporting for specific materials, systems and methods

We require information from authors about some types of materials, experimental systems and methods used in many studies. Here, indicate whether each material, system or method listed is relevant to your study. If you are not sure if a list item applies to your research, read the appropriate section before selecting a response.

Materials & experimental systems

n/a	Involved in the study	
☐	☒	Antibodies
☒	☐	Eukaryotic cell lines
☒	☐	Palaeontology and archaeology
☒	☐	Animals and other organisms
☒	☐	Clinical data
☒	☐	Dual use research of concern

Methods

n/a	Involved in the study	
☒	☐	ChIP-seq
☒	☐	Flow cytometry
☒	☐	MRI-based neuroimaging

Antibodies

Antibodies used	anti-CsoS1A/B/C (Agrisera, Cat No. AS14 2760, 1: 5,000 dilution), anti-CsoS2-N (synthesized by GenScript, USA, 1:10,000 dilution)
Validation	The antibody of anti-CsoS1A/B/C is a commercial antibody and has been widely used in our and others' Labs. Home-made anti-CsoS2-N antibody was produced by Genscript and can be specific differentiate the target protein form the samples.
